# The effect of the great Kermanshah earthquake on the tuberculosis infection trend: interrupted time series analysis

**DOI:** 10.1186/s12879-024-09832-w

**Published:** 2024-11-07

**Authors:** Ehsan Mardani-Talaee, Yahya Salimi, Fatemeh Heydarpour, Mitra Darbandi, Gholamreza Abdoli

**Affiliations:** 1 Student Research Committee, Kermanshah University of Medical Sciences, Kermanshah, Iran; 2 School of Public Health, Kermanshah University of Medical Sciences, Kermanshah, Iran

**Keywords:** Earthquake, Tuberculosis, Interrupted time series

## Abstract

**Objectives:**

Tuberculosis (TB) presents a substantial danger to populations experiencing crises like earthquakes. This study aims to explore the effect of the Kermanshah earthquake on the trend of TB.

**Methods:**

This cross-sectional study examined tuberculosis data from 2009 to 2020, using monthly diagnoses. Data was collected from the TB research office and registration system. The study employed interrupted time series (ITS) analysis to assess both the immediate and long-term impacts of the earthquake on TB cases.

**Results:**

In Sarpol-e Zahab, TB cases initially surged after the earthquake, indicating an immediate effect, but then significantly declined compared to pre-earthquake levels, reflecting an effect over time (β_0_ = 1.39, β_1_=-0.004, β_2_ = 0.11 and β_3_=-0.01, *P* = 0.001 and Post-intervention linear trend= -0.015, *P* < 0.001). In Ghasr-e Shirin, the average number of TB cases prior to the earthquake was estimated at 0.58 cases, with a significant monthly decrease of 0.005 cases leading up to the earthquake (*P* = 0.001). There was no significant immediate change in TB cases during the first month after the earthquake (β_2_ = 0.008, *P* = 0.680). Post-earthquake, TB cases dramatically increased (β_3_ = 0.008, *P* = 0.001). The monthly trend of TB cases rose significantly by 0.002 (*P* = 0.001), indicating an effect over time. In Salas-e Babajani, there was no immediate change in TB cases, but there was a significant long-term decline compared to the period before the earthquake (*P* = 0.001).

**Conclusions:**

Earthquake is one of the natural crises that provide the conditions for the increase of TB. Local health policymakers must make plans in these areas to contain TB after the earthquake.

## Introduction

Every year, 10 million individuals are diagnosed with tuberculosis (TB). Although it is a disease that can be prevented and treated, 1.5 million people lose their lives to TB annually [1]. The TB incidence rate in Iran has followed a downward trend over the last 50 years [2]. In 2018, the incidence rate of all TB manifestations including pulmonary TB, both positive and negative smears, and extra-pulmonary TB decreased from 142 cases in 1964 to 61 cases per 100,000 population, which decreased more than 13 times. Meanwhile, the incidence rate of the disease in Kermanshah Province was reported as 6.72 cases per 100,000 people (2.12 extrapulmonary, 1.52 negative, and 2.73 positive smears) [3]. Most infections brought about by natural disasters are commonly caused by the indigenous microbes as 14% of indigenous inhabitants contracted respiratory diseases after the Bam earthquake [4]. previous studies show that death rate from TB after earthquakes has increased. Strict adherence to the full TB treatment is necessary. However, the extensive excavations and the damage done by the emergency make it difficult to continue the treatment of patients with TB [5–7].

The provision of healthcare services after an earthquake differs significantly from normal conditions. The 7.3 magnitude earthquake that struck Kermanshah on November 12, 2017, at a depth of 23 km, severely affected living conditions for an extended period, particularly in the cities of Qasr-e Shirin, Sarpol-e Zahab, and Salas-e Babajani. Access to basic health needs became a major concern, potentially facilitating the spread of infectious diseases. This study aims to investigate changes in tuberculosis trends during the earthquake using Interrupted Time Series (ITS) analysis.

## Method

### Study design

The current study is a cross-sectional study that compares the pattern of TB occurrence 8 years before and three years after the earthquake in Kermanshah province. The statistical population of all TB cases occurred in the residents of the earthquake-affected areas in the three cities of SarPol-e Zahab, Ghasr-e Shirin and Salas-e Babajani in Kermanshah province in the west of Iran. The researchers used the census sampling method, which means they studied all patients with pulmonary and extrapulmonary tuberculosis infection in the community.

The inclusion criteria for the study included living in earthquake-affected areas from 2007 to three years after the earthquake. The study included patients of all ages, considering both pulmonary and extrapulmonary tuberculosis cases. The only exclusion criterion was if a patient did not have sufficient clinical. The diagnosis method was confirmed by two sputum smear samples in all patients.

Data on TB patients was collected from various sources:

I. Pulmonary centers affiliated with the Health Department of Kermanshah Province (https://tb.behdasht.gov.ir/).
II. Healthcare centers linked to the University of Medical Sciences in Kermanshah.
III. Private medical facilities in Kermanshah province.

### Statistical analysis

There was a lag (1) in the adjustment of the initial model due to the autocorrelation in the lag 1 for different series of the disease including the type of disease and disease results, and to make sure the estimation of the model taking into account the right correlation structure, lag (6) was used to investigate the autocorrelation and to select the best lags to estimate the standard regression model (ITS) in the following way:

$$Yt\, = \,{\beta _0}\, + \,{\beta _1}\,{T_t}\, + \,{\beta _2}\,{X_t}\, + \,{\beta _3}\,{X_t}\,{T_t}\, + \,\varepsilon t$$

Y_*t*_: The sum of cases that occurred at the same time distances at any time point.

T_t_: Since the beginning of the study.

X_t_: A categorical variable showing the intervention (periods before the intervention 0, others 1).

X_t_T_t_: An interaction term.

β_0_: Shows the intercept i.e. the surface from which the dependent variable starts.

β_1_: Consequence variable slope before the intervention (earthquake).

β_2_: The change in the level of the result occurring immediately at the start of the intervention (compared to the unreal).

β_3_: The difference between the consequence slope before and after the intervention.

β2 has an immediate effect and β3 has an effect over time. The TB cases before the earthquake were considered a baseline (control period) for the city.

**Fig. 1:**
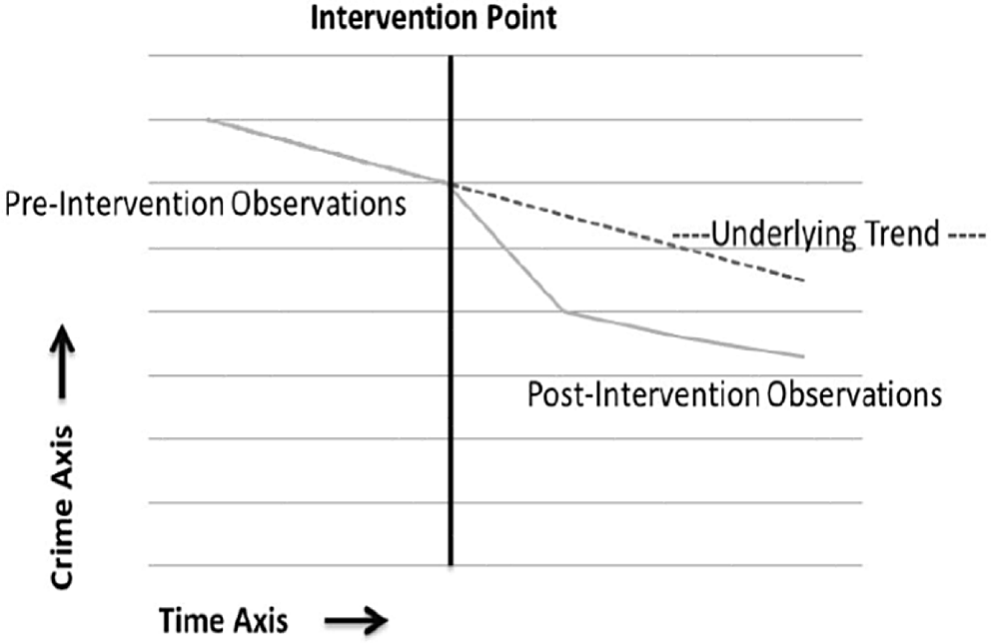
Interrupted time series analysis

To assess how the earthquake affected tuberculosis case numbers, we employed an Interrupted Time Series (ITS) analysis with segmented regression using ordinary least squares. Additionally, we applied Newey-West standard errors to account for potential heteroscedasticity and autocorrelation. For the time series analysis, we organized the variables as follows: (1) the overall count of TB cases, including subcategories for pulmonary and extra-pulmonary tuberculosis; (2) the earthquake variable, serving as the intervention factor, recorded as zero before the earthquake and one afterward; (3) a time variable representing the study duration, measured in months from one to 131 (Fig. 1).

All statistical analyses for this study with statistical significance set at less than 0.05 and 95% confidence interval (CI) were carried out using Stata statistical software version 14.2 (Stata Corp, College Station, TX, USA).

## Results

A total of 132 TB cases were analyzed in Sarpol-e Zahab city, with 117 cases reported before the earthquake and 15 cases afterward. The average number of initial TB in a stable condition (before the earthquake) was estimated to be 1.39 cases. The number of TB cases significantly decreased by 0.004 every month until the occurrence of the earthquake on 12 November 2017 (P-value for baseline trend = 0.001). In the first month after the earthquake, there was a notable rise in TB cases by 0.11 (*P* = 0.001, 95% CI: 0.04–0.18), indicating an immediate effect. Moreover, in comparison to the time before the earthquake, a significant reduction in the monthly trend of tuberculosis cases was noted, estimated at 0.01 cases (*P* = 0.001, 95% CI:-0.008 to -0.01). Following the earthquake, the monthly trend (effect over time) of TB cases continued to decline significantly at a rate of 0.015 (*P* = 0.001, 95% CI: -0.01 to -0.02) (Table 1).

**Table 1:** Estimation of segmented regression model coefficients for the total number of tuberculosis patients in earthquake-affected counties from March 2009 to February 2020

County	Total number of tuberculosis cases	Model coefficient		Standard deviation	t	p>|t|	Confidence interval 95%	
							lower	upper
**Sarpol- e Zahab**	Intercept	β_0_	1.390	0.033	41.23	0.001	1.460	1.330
	Pre-intervention consequence slope	β_1_	-0.004	0.0004	-8.30	0.001	-0.003	-0.005
	Impact of rapid change in level	β_2_	0.110	0.030	3.49	0.001	0.180	0.040
	Interplay between time and intervention	β_3_	-0.010	0.001	-9.01	0.001	-0.008	-0.010
	Post-intervention linear trend	-0.015		0.001	-13.46	0.001	-0.013	-0.017
	Regression using standard deviation Wewey-West; Maximum lag:1; Number of observations = 131; F(3 and 127) = 216.94; Prob > F = 0.001.							
**Ghasr- e Shirin**	Intercept	β_0_	0.589	0.031	18.84	0.001	0.65	0.52
	Pre-intervention consequence slope	β_1_	-0.005	0.0004	-12.55	0.001	-0.004	-0.006
	Impact of rapid change in level	β_2_	0.008	0.019	0.41	0.680	0.047	-0.031
	Interplay between time and intervention	β_3_	0.008	0.0008	10.11	0.001	0.009	0.006
	Post-intervention linear trend	0.0025		0.0007	3.71	0.001	0.001	0.004
	Regression using standard deviation Wewey-West; Maximum lag:1; Number of observations = 127; F(3 and 123) = 114.84; Prob > F = 0.001.							
**Salas-e Babajani**	Intercept	β_0_	0.40	0.00004	8950.06	0.001	0.4066	0.4064
	Pre-intervention consequence slope	β_1_	-4.46e^− 6^	8.67	-5.15	0.001	-2.74e^− 6^	-6.18e^− 6^
	Impact of rapid change in level	β_2_	0.0001	0.00006	1.80	0.075	0.0002	-0.00001
	Interplay between time and intervention	β_3_	-0.00001	2.36	-7.77	0.001	-0.0001	-0.00002
	Post-intervention linear trend	-0.0000		0.00	-10.41	0.001	-0.0000	-0.00
	Regression using standard deviation Wewey-West; Maximum lag:1; Number of observations = 129; F(3 and 125) = 113.31; Prob > F = 0.001							

For Ghasr-e Shirin County, 29 patients with TB, 26 cases before and three cases after the earthquake, were examined. The average number of TB cases in a stable state (prior to the earthquake) was estimated to be 0.58 cases, and the number of TB cases significantly decreased 0.005 every month until the earthquake struck (P-value for baseline trend = 0.001).

We did not detect any significant immediate change in TB cases during the first month after the earthquake, indicating no immediate effect. However, compared to the period before the earthquake, the number of TB cases increased dramatically after the earthquake (*P* = 0.001). Additionally, following the earthquake, the monthly trend of TB cases rose significantly by 0.002, indicating an effect over time (*P* = 0.001, 95% CI = 0.001 to 0.004).

The data for 41 cases before and five cases after the earthquake were included for evaluation of trend TB in Salas-e Babajani County. The average number of TB cases in a stable state (prior to the earthquake) was estimated to be 0.40 cases. The number of TB cases significantly declined up to the earthquake time (P-value for baseline trend = 0.001). In the first month following the earthquake, no significant increase was noted in the level, suggesting no immediate effect. However, the number of TB cases declined sharply after the earthquake compared to the period before, indicating an effect over time (*P* = 0.001).

**Fig. 2:**
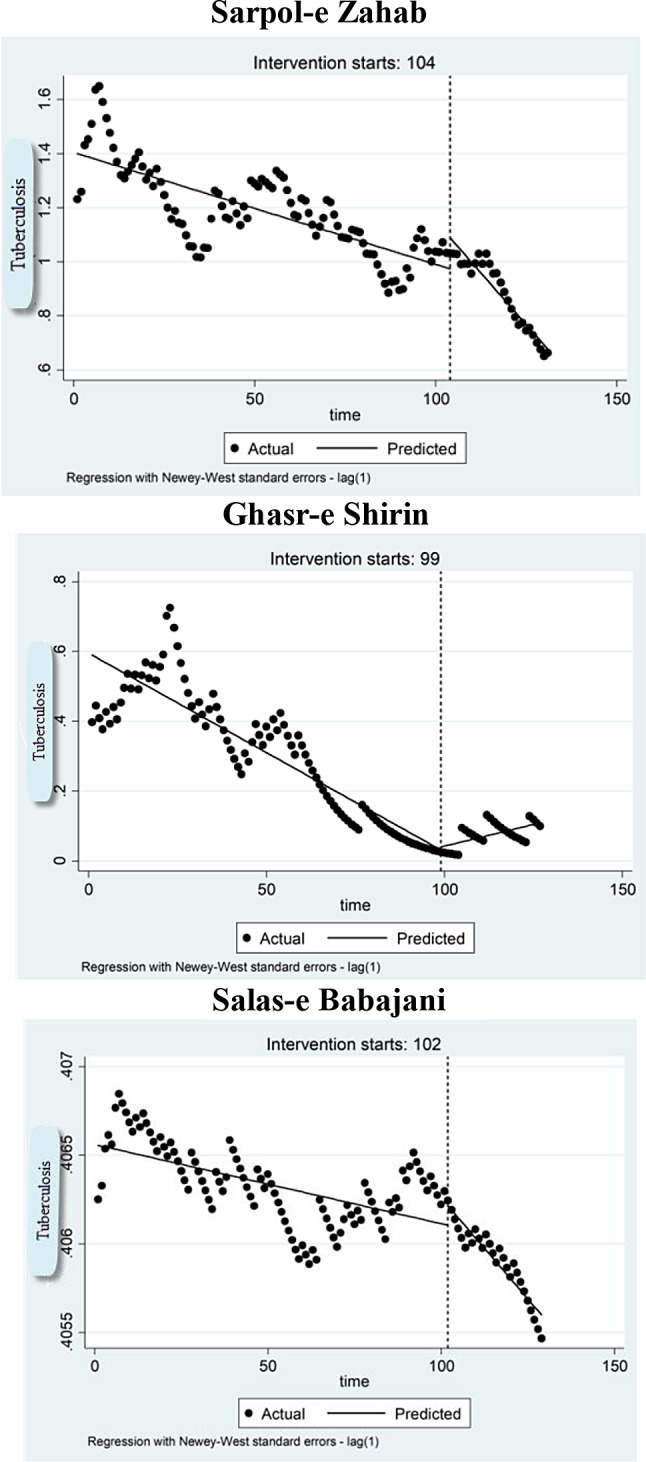
Changes in the total number of tuberculosis patients before and after the earthquake in Sarpol-e Zahab, Ghasr-e Shirin and Salas-e Babajani

Figure 2 shows the trend of TB cases in earthquake-affected counties from March 2009 to February 2020.

## Discussion

This study evaluated the impact of the 7.3-magnitude Kermanshah earthquake on TB trends in the three most affected cities: Sarpol-e Zahab, Ghasr-e Shirin, and Salas-e Babajani. In Sarpol-e Zahab, TB cases initially increased after the earthquake, indicating an immediate effect, but later decreased compared to pre-earthquake levels, reflecting an effect over time. In Ghasr-e Shirin, the trend of TB cases declined leading up to the earthquake, and while there was an increase in cases afterward, this change was not statistically significant. However, the trend following the earthquake showed a significant upward slope compared to the pre-intervention period, indicating an effect over time. Conversely, Salas-e Babajani experienced a consistent decrease in TB cases without any notable changes after the earthquake.

The study by Koenig and et al. indicates that there has been a rise in reported TB cases in Haiti after the earthquake. However, the data is too limited to establish whether this increase stems from a greater burden of TB or from better detection methods. Although active case-finding initiatives have successfully identified more TB patients, the current scale of these efforts does not sufficiently account for the observed rise in cases [8]. Research conducted in China has revealed that several factors impact the incidence of pulmonary tuberculosis during and after an earthquake. These factors include the presence of minority populations, the percentage of the workforce, the city’s latitude, and the earthquake’s intensity. Over time, the influence of minority areas, occupational factors, and health resource distribution has diminished, while economic development and population mobility have become increasingly important [9]. Yang et al.‘s study in the area affected by the Wenchuan earthquake found a consistent trend without post-earthquake ruptures from 2004 to 2012, showing a decline in occurrence from north to south across Sichuan Province [10]. A study examining the health conditions in the cities of Kermanshah Province affected by the earthquake revealed that the health and treatment system suffered significant damage. Issues identified include inadequate case registration, poor management of health concerns, and insufficient patient tracking, despite the establishment of patient guidance centers [11]. The discrepancies found in the study results may stem from incomplete reporting following the earthquake, which was caused by disruptions in the public health centres (PHCs). Furthermore, many patients may have avoided visiting health centers due to trauma and depression resulting from the earthquake. Another factor could be the reliance on limited or varied data sources across different studies. For instance, the research conducted in Haiti utilized a tuberculosis data source that may not accurately reflect changes in prevalence [8]. In contrast, this study employed three data sources, enhancing the reliability of the results.

In the research conducted by Kanamori et al., which investigated tuberculosis transmission following the 2011 earthquake and tsunami in Japan, several risk factors for tuberculosis prognosis were identified. These include advanced age, low serum albumin levels, functional status during hospitalization, and the requirement for oxygen. Additionally, the findings from Variable Number of Tandem Repeats (VNTR) analysis indicate that most pulmonary tuberculosis cases were linked to the reactivation of latent tuberculosis infections, likely triggered by the earthquake and tsunami’s effects [12].

In Ghasr-e Shirin, the trend of TB cases showed a decline leading up to the earthquake. After the earthquake, there was an increase in cases, although this change was not statistically significant. However, the trend of cases following the earthquake demonstrated a significant upward slope compared to the pre-intervention period. Follow-up studies on TB infections in the northern coastal region of Miyagi after the Great East Japan Earthquake revealed that in the early phase (2011–2012), there was a notable increase in the total number of TB patients, as well as cases of pulmonary tuberculosis and latent TB infection (LTBI) specifically in the coastal areas severely impacted by the tsunami. However, during the intermediate phase (2013–2014), although there was a significant decline in the overall cases of TB, pulmonary TB, and LTBI, their prevalence had not returned to the levels seen before the disaster. These findings indicate that the effects of the Great East Japan Earthquake continue to persist [13]. The rapid increase in cases can stem from the increase in relief services and the presence of healthcare staff dispatched from other areas during the period when the damage is at its most severe point, which leads to the diagnosis of a larger number of TB cases. In other words, earthquakes do not increase TB cases over a short period of some weeks; thus, the downward trend in the long run compared to the period before the earthquake may originate from the poor management of the disease.

Previous studies show after the earthquake, there has been a significant increase in TB and latent TB infections, particularly among individuals in crowded shelters. Key factors contributing to this rise include power and water outages, road closures affecting access to food and medical care, and the destruction of healthcare facilities [14–16]. Additionally, lower intake of essential nutrients during crises increases the risk of TB progression and mortality. Overcrowding in shelters underscores the need for effective preparation to prevent congestion [17, 18]. A strong healthcare system is vital for TB control in disaster situations, yet disruptions in services can worsen the disease and accelerate transmission [19]. Earthquakes also lead to conditions like stress, malnutrition, and poor sanitation, which can increase smoking behaviors, further heightening the risk of TB infection and progression [20–24] .

The trend of TB in Sarpol-e Zahab, Qasr-e Shirin, and Salas-e Babajani post-earthquake may have varied due to differences in healthcare infrastructure damage, access to medical services, and healthcare-seeking behavior influenced by psychological trauma. Social and economic conditions also affected responses to health crises, while discrepancies in data quality could lead to inconsistencies in reported prevalence.

Accessing data at appropriate time intervals for conducting an ITS analysis was a notable strength of this study. However, during disasters such as earthquakes, routine patient registration programs may be disrupted, leading to an undercount of cases. Additionally, there was relatively insufficient diagnosis of tuberculosis and incomplete recording of patient information at the centers involved in the study. This could have resulted in incomplete or inaccurate data collection.

## Conclusion

After the Kermanshah earthquake, TB cases in Sarpol-e Zahab initially surged significantly but later decreased notably compared to pre-earthquake levels. In Ghasr-e Shirin, there was a decline in TB cases leading up to the earthquake, followed by an increase afterward that was not statistically significant; however, the trend post-earthquake showed a significant upward slope. In contrast, Salas-e Babajani experienced a steady decrease in tuberculosis cases without any abrupt changes after the earthquake. This highlights the need for healthcare policymakers to develop a resilient system to effectively manage tuberculosis in challenging conditions, such as those following an earthquake.

## Data Availability

The data analyzed in the study are available from the corresponding author upon reasonable request.

## Abbreviations

ITS: Interrupted time series
TB: Tuberculosis
CI: Confidence interval
PHCs: Public health centres
VNTR: Variable Number of Tandem Repeats
LTBI: Latent TB infection
